# Seventy FDG-PET/CT Cases in Which Nuclear Medicine Physicians Suspected Lymphoma: How Reliable Are We?

**DOI:** 10.22038/aojnmb.2017.8767

**Published:** 2017

**Authors:** Akira Toriihara, Masashi Nakadate, Shin Nakamura, Kazunori Kubota, Ukihide Tateishi

**Affiliations:** 1 Department of Diagnostic Radiology and Nuclear Medicine, Tokyo Medical and Dental University, Tokyo, Japan; 2 Department of Oral and Maxillofacial Radiology, Tokyo Medical and Dental University, Tokyo, Japan; 3 Department of Medical Informatics, Tokyo Medical and Dental University, Tokyo, Japan

**Keywords:** FDG-PET/CT, Lymphoma, Soluble interleukin-2 receptor, Spleen, SUV_max_

## Abstract

**Objective(s):**

To validate the reliability of nuclear medicine physicians in diagnosing lymphoma using positron emission tomography/computed tomography using 2-deoxy-2-[^18^F]fluoro-D-glucose (FDG-PET/CT) and to determine findings that reliably suggest lymphoma.

**Methods:**

Seventy patients suspected of having lymphoma using FDG-PET/CT were enrolled in this retrospective study. Two nuclear medicine physicians read all the interpretation reports and graded the degree of suspicion by consensus (3: definitely suspicious, 2: probably suspicious, and 1: possibly suspicious). The following factors were also investigated for each patient: maximum standardized uptake value (SUV_max_) of the lesions, serum level of soluble interleukin-2 receptor (sIL-2R), and the presence of splenic FDG uptake higher than that of the liver.

**Results:**

The study group consisted of 34 lymphomas, 18 other malignancies, and 18 benign lesions according to histopathological diagnosis. No patient with a Grade 1 degree of suspicion was diagnosed as lymphoma. SUV_max_ and the serum level of sIL-2R could not distinguish lymphoma from other diseases. Of the 11 patients who presented with elevated splenic FDG uptake, 10 were diagnosed as having lymphoma.

**Conclusion:**

When the degree of suspicion by nuclear medicine physicians is low, the possibility of lymphoma is also low. On the other hand, elevated splenic FDG uptake may suggest lymphoma.

## Introduction

Malignant lymphoma is a relatively common malignancy affecting all age groups (1). Invasive methods, such as biopsy, are required for a definitive diagnosis of lymphoma. Radiologists and nuclear medicine physicians should be familiar with the imaging findings of lymphoma to make an appropriate clinical diagnosis. However, lymphoma can involve every tissue in the body and their imaging findings vary widely (1, 2). Therefore, imaging findings can be misinterpreted, leading to misdiagnosis, as well as false-positive or false-negative results.

Recently, the usefulness of positron emission tomography/computed tomography using 2-deoxy-2-[^18^F]fluoro-D-glucose (FDG-PET/CT) has been established for lymphoma management. Applications of FDG-PET/CT include staging, monitoring therapy, and prognosis of lymphoma that has been definitely diagnosed (3, 4). However, FDG-PET/CT has a limitation for differential diagnosis because other malignant neoplasms or active inflammatory diseases also show elevated FDG uptake (5, 6).

As the use of FDG-PET/CT has become more widespread in oncology, nuclear medicine physicians often encounter cases in which lymphoma is suspected in patients undergone PET/CT for another reason. There has been a wide range of case reports describing the difficulty in distinguishing lymphoma from other diseases (7-11). Nonetheless, to the best of our knowledge, no studies have been conducted to investigate the potential of FDG-PET/CT in differential diagnosis in patients with suspected lymphoma. Although a biopsy is needed for diagnosis of lymphoma, it is an invasive method and should be ideally limited to patients with highly probable suspicion. Understanding FDG-PET/CT findings, which strongly suggest the possibility of lymphoma, can contribute to appropriate patient management.

Our objectives were to review FDG-PET/CT studies where nuclear medicine physicians suspected lymphoma in interpretation reports and to determine findings that reliably suggest lymphoma.

## Methods

Our institutional review board approved this retrospective study, and the need for written informed consent was waived (IRB approval number: 2025).

### Patients

We reviewed 6,962 FDG-PET/CT studies performed at our hospital from September 2010 to December 2012. Studies in which lymphoma was suspected in their interpretation reports were selected. All the reports were completed by consensus of two or three evaluators including both radiologists and nuclear medicine physicians. In all the PET/CT studies, at least one nuclear medicine physician with prior experience of evaluating over 2,000 PET/CT studies participated in editing interpretation reports.

We found 183 PET/CT studies in which the possibility of lymphoma was stated in their interpretation reports. We excluded 113 cases based on the following criteria: 1) history of lymphoma or other malignant neoplasms, 2) histopathological procedure (cytology or biopsy) before FDG-PET/CT suggested any malignant neoplasms including lymphoma, and 3) final diagnosis unconfirmed by histopathology. Finally, 70 patients (36 males and 34 females, with mean age of 62.4 years) were enrolled in this retrospective study. More than half of them (37 patients) had undergone FDG-PET/CT for investigation of lymphadenopathy of unknown cause. Other referral indications for FDG-PET/CT were solid tumor in various organs in 25 patients, splenomegaly in two patients, elevated soluble interleukin-2 receptor (sIL-2R) in one patient, and other objectives in five patients.

According to the final diagnosis, all the patients were divided into 1) group L (lymphoma), 2) group M (other malignancies), and 3) group B (benign diseases).

### FDG-PET/CT protocol

After fasting for at least 4 h, the patients received an intravenous injection of ^18^F-FDG (3.7 MBq/kg). Sixty minutes after the FDG injection, whole-body PET/CT images were obtained using a PET/CT system (Aquiduo, Toshiba Medical Systems, Tokyo, Japan) consisting of a combination of a full-ring PET scanner with lutetium oxyorthosilicate crystals and a 16-row helical CT scanner. CT studies for attenuation correction were performed under expiratory breath-holding using the following parameters: 120 kV; 50-200 mAs, field of view 500 mm, pitch 15.0, and slice thickness of 2.0 mm.

Under free breathing, acquisition of PET emission data was completed using the following parameters: 3D mode with scan time of 2 min per bed position (for 6-8 bed positions), matrix size of 128×128, and Gaussian filter size of 5 mm. All the patients drank approximately 300 ml of water after the FDG injection for oral hydration and gastric dilatation. The subjects were instructed to evacuate their bladder just before the PET/CT imaging. No contrast media was used.

### Grading degree of suspicion of lymphoma

Two nuclear medicine physicians, who were different from the writers of interpretation reports, read all the reports and graded the degree of suspicion by consensus (Grade 3: definitely suspicious, Grade 2: probably suspicious, and Grade 1: possibly suspicious). Grading was performed mainly based on the number of differential diagnoses referred to in each report. We listed the definition for each grade in Table 1.

**Table 1:** Grade criteria for degree of suspicion of lymphoma

Grade	Reports
3: Definitely suspicious	No other diseases were referred to in the report.
2: Probably suspicious	One or two diseases, except for lymphoma, were suggested as differential diagnoses. AND The evaluators suspected lymphoma in a degree equal to or greater than other diseases.
1: Possibly suspicious	More than three differential diagnoses, except for lymphoma, were proposed. OR Even if one or two differential diagnoses were proposed, the evaluators clearly suspected other diseases compared to lymphoma.

### Image analysis

FDG-PET/CT images of the enrolled patients were retrospectively evaluated by the same two nuclear medicine physicians. The maximum standardized uptake value (SUV_max_) was calculated for one lesion, regardless of nodal or extranodal, with the highest FDG uptake. Because our study focused on the possibility of lymphoma, we also judged whether FDG uptake in the spleen, which is highly involved in lymphoma, exceeded FDG uptake in the liver by visual interpretation (12). If sIL-2R, often used as a tumor marker of lymphoma, was measured one month before or after FDG-PET/CT study, we reviewed its serum level for this study.

### Statistical analysis

The Kruskal-Wallis test was performed to assess the difference between the three groups (L, M, and B) in terms of SUV_max_ and sIL-2R. If statistical significance (P<0.05) was confirmed, Mann-Whitney test was performed between pairs of groups, including L vs M, L vs B, and M vs B, respectively. Using Bonferroni’s adjustment, P<0.016 was considered as the cut-off for statistical significance.

## Results

**Figure 1:**
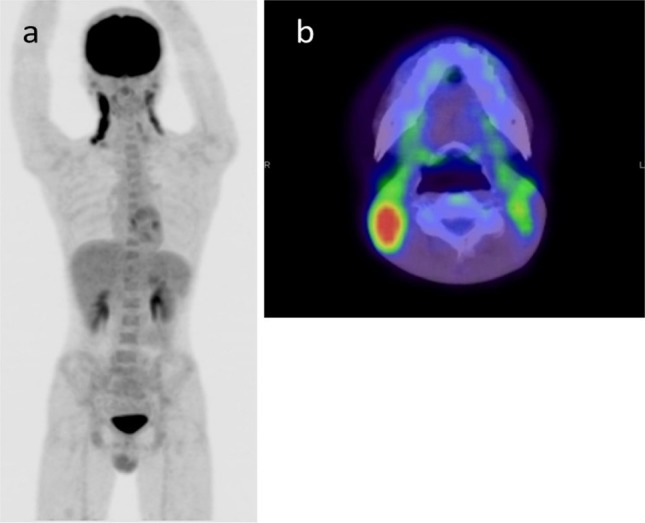
A 21-year-old man with Kikuchi’s disease misdiagnosed as lymphoma; (a) Maximum intensity projection image and (b) fused FDG-PET/CT image of the neck are shown. The patient presented with cervical lymphadenopathy and a fever of 39°C for a month. FDG-PET/CT showed bilateral cervical lymphadenopathy with elevated FDG uptake (SUV_max_=8.7). Elevated sIL-2R of 1200 U/ml was also observed. Although the evaluators commented that this case was compatible with lymphoma (degree of suspicion was Grade 3), the final biopsy-proven diagnosis was Kikuchi’s disease

**Figure 2:**
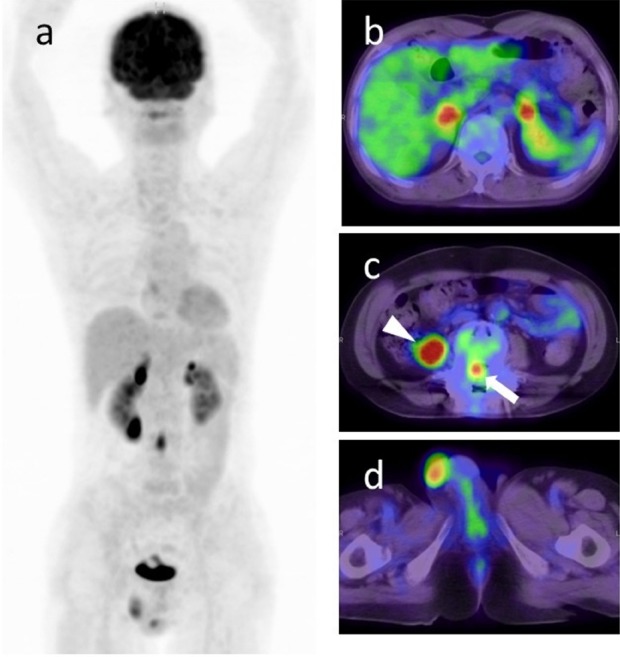
A 59-year-old man with lymphoma; (a) Maximum intensity projection image and (b-d) fused FDG-PET/CT images are shown. The patient presented with motor and sensory disturbance of the bilateral lower limbs for six months. FDG-PET/CT showed multiple lesions in bilateral adrenal glands (b) (SUV_max_=17.1 in the right, and 8.8 in the left), cauda equine (c; white arrow) (SUV_max_=7.9 at the level of L3), right retroperitoneum (c; white arrowhead) (SUV_max_=14.3), and bilateral testes (d) (SUV_max_=6.1 in the right, and 4.9 in the left) with elevated FDG uptake. Elevated sIL-2R of 1680 U/ml was also observed. Given the distribution and extremely elevated FDG uptake of lesions, the evaluators commented that lymphoma was most strongly suspected. On the other hand, although they considered that a case without any nodal metastases but with distant metastases as stated above is very rare, metastatic testicular tumor was also added to differential diagnosis (degree of suspicion was Grade 2). Right orchiectomy was performed and confirmed diagnosis was diffuse large B-cell lymphoma

**Figure 3:**
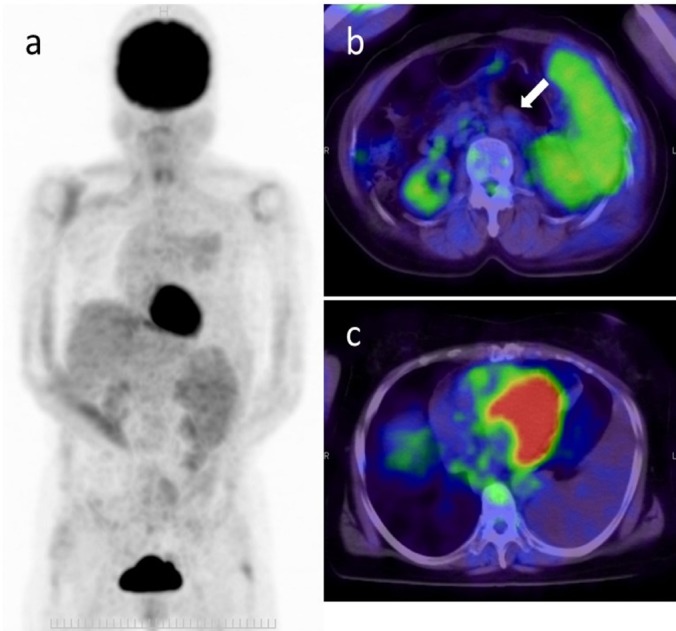
A 59-year-old woman with lymphoma; (a) Maximum intensity projection image and (b, c) fused FDG-PET/CT images are shown. The patient was referred to our hospital for further evaluation and treatment of left pleural effusion, splenomegaly, and paraaortic lymphadenopathy. The level of sIL-2R was within normal limit (378 U/ml). FDG-PET/CT showed splenomegaly with diffusely increased FDG uptake (SUV_max_=3.9) (b) and left pleural effusion (c). Although paraaortic lymphadenopathy was also observed (b; white arrow), the FDG uptake was mild (SUV_max_=1.8). The evaluators commented that this case was compatible with low-grade lymphoma. Confirmed diagnosis obtained by splenectomy was peripheral T cell lymphoma

The final diagnoses of 70 patients are presented in Table 2. Groups L, M, and B comprised of 34 (48.6%), 18 (25.7%), and 18 (25.7%) patients, respectively. We illustrated representative cases in Figures 1-3.

**Table 2:** Confirmed diagnoses in 70 patients

Group	Diagnosis	Number
Lymphoma (Group L)	Diffuse large B cell lymphoma	15
	Follicular lymphoma	5
	Hodgkin lymphoma	4
	T cell lymphoma (unknown)	3
	Peripheral T cell lymphoma	2
	Extranodal NK/T cell lymphoma, nasal type	1
	Plasmablastic lymphoma	1
	B cell lymphoma (unknown)	1
	Angioimmunoblastic T cell lymphoma	1
	Myeloid/NK cell precursor acute leukemia	1
Other malignancy (Group M)	Carcinoma (primary unknown)	7
	Lung cancer	5
	Cancer arising from ectopic pancreas	1
	Thyroid cancer	1
	Malignant melanoma	1
	Appendiceal cancer	1
	Gastric cancer	1
	Prostate cancer	1
Benign lesion (Group B)	Sarcoidosis	3
	Follicular hyperplasia	3
	Tuberculous lymphadenitis	2
	Syphilitic lymphadenitis	2
	Kikuchi’s disease	2
	Castleman’s disease	2
	Fibrinous pleuritic	1
	IgG4-related disease	1
	*Mycobacterium avium* complex infection	1
	Severe inflammation (cause unknown)	1

### The relationship between the degree of suspicion of lymphoma and final diagnoses

We demonstrated the relationship between the degree of suspicion of lymphoma and the confirmed diagnoses in Table 3. There were six false-positive cases (21.4%), in whom lymphoma was definitely suspected (Grade 3) by nuclear medicine physicians (one Kikuchi’s disease, one IgG4-related disease, two syphilitic lymphadenitis, one tuberculous lymphadenitis, and one severe inflammation).

**Table 3:** Relationship between confirmed diagnosis and degree of suspicion of lymphoma

	Degree of suspicion			
	Grade 3	Grade 2	Grade 1	Total
Confirmed diagnosis				
Group L	22	12	0	34
Group M	0	9	9	18
Group B	6	7	5	18
Total	28	28	14	70

Group L, lymphoma; Group M, other malignancy; Group B, benign lesion

No patient was assessed as Grade 3 in the group M. None of the patients assessed as Grade 1 received a confirmed diagnosis of lymphoma.

### FDG-PET/CT findings and sIL-2R

**Figure 4:**
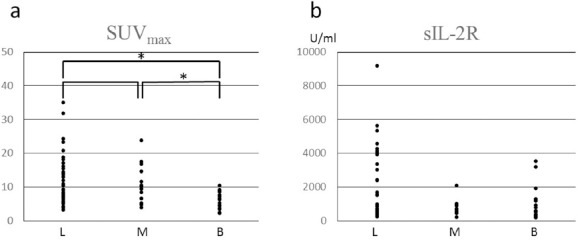
SUV_max_ and sIL-2R in three groups; a: There was a significant difference in SUV_max_ between the three groups (P=0.002; Kruskal-Wallis test). Mann-Whitney test revealed that the groups L and M showed significantly higher SUV_max_ than that of group B (P<0.05^*^). b: There was no significant difference in sIL-2R between the three groups (P=0.068; Kruskal-Wallis test). Note: Group B, group of benign diseases; Group L, group of lymphoma; Group M, group of other malignancies; sIL-2R, soluble interleukin-2 receptor; SUV_max_, maximum standardized uptake value

Figure 4a illustrates the distribution of SUV_max_ in each case. Means of SUV_max_ were 12.3±7.8, 10.3±5.2, and 6.1±2.6 in the groups L, M, and B, respectively. The Kruskal-Wallis test revealed a significant difference in SUV_max_ between the three groups (P=0.002). Groups L and M showed significantly higher SUV_max_ than that observed in group B (P=0.001 and P=0.003, respectively). There was no significant difference in SUV_max_ between groups L and M (P=0.56). We showed SUV_max_ of each subtype of lymphoma in Table 4. Although we did not perform statistical analysis because of the limited sample size, there was a tendency that mature B cell neoplasms, especially diffuse large B cell lymphoma, show higher SUV_max_ compared to other subtypes.

**Table 4:** Maximum standardized uptake value of each lymphoma subtype

Subtype	Number	SUV_max_
<B cell>		
Diffuse large B cell lymphoma	15	17.3±8.1
Follicular lymphoma	5	6.4±0.9
Plasmablastic lymphoma	1	10.5
B cell lymphoma (unknown)	1	18.1
<T- or NK-cell>		
Peripheral T cell lymphoma	2	3.6±0.5
Angioimmunoblastic T cell lymphoma	1	4.2
T cell lymphoma (unknown)	3	10.0±2.6
<Others>		
Hodgkin lymphoma	4	6.5±1.4
Myeloid/NK cell precursor acute leukemia	1	9.3

SUV_max_, maximum standardized uptake value (mean±standard deviation)

Eleven patients presented splenic FDG uptake higher than that observed in the liver. Of these, 10 patients (90.9%) had a confirmed diagnosis of lymphoma. The last patient was diagnosed as Castleman’s disease.

There were 60 patients (85.7%) who underwent measurement of sIL-2R within a month from each PET/CT study. Means of sIL-2R were 2064.2±2089.7 U/ml, 777.8±498.2 U/ml, and 1023.9±1029.0 U/ml in the groups L (33 patients), M (11 patients), and B (16 patients), respectively. There was no significant difference in sIL-2R between the three groups (*P*=0.068; Figure 4b).

## Discussion

We sometimes encounter FDG-PET/CT cases in which it is difficult to discriminate lymphoma from other diseases. However, using the specific findings from FDG-PET/CT, grading the possibility of lymphoma facilitates better management, specifically by allowing physicians to suggest the necessity of an invasive procedure such as a biopsy. Therefore, we selected FDG-PET/CT findings, which strongly suggested lymphoma in this study.

We evaluated the relationship between the confirmed diagnosis and the degree of suspicion of lymphoma as determined by interpretation reports. There were six cases that were proved to be benign lesions even though nuclear medicine physicians strongly suspected lymphoma (Grade 3). As also shown in Figure 1, some kinds of benign diseases can often show elevated FDG uptake similar to malignant tumors (7-11). On the other hand, no patient meeting the Grade 1 criteria was diagnosed with lymphoma. Because FDG-PET/CT findings associated with lymphoma are quite variable, the evaluators cannot diagnose lymphoma with certainty but instead can suggest the possibility of lymphoma using FDG-PET/CT as part of a differential diagnosis. However, readily suggesting lymphoma in Grade 1 cases may not only result in misdiagnosis but also in loss of confidence in the diagnostic capacity by clinicians and patients.

No patient was evaluated as Grade 3 in the group M. The evaluators may have decreased the suspicion of lymphoma, considering the lymph node distribution and pattern of FDG-avid lesions, and consequently avoided misdiagnosis. In this retrospective study, there were no consensus rules to differentiate between lymphoma and other malignant tumors. Therefore, further prospective studies, according to the common criteria for lymphoma diagnosis, are required to confirm the relationship between the distribution or pattern of diseases and the final confirmed diagnosis.

We found that lymphoma and other malignancies show higher SUV_max_ than benign diseases. However, active inflammatory diseases also show elevated FDG uptake. As Figure 4A reveals, there were some overlaps between malignant tumors and benign diseases, particularly in the range of SUV_max_ lower than 10. Furthermore, there was variability of SUV_max_ among different types of lymphoma as shown in Table 4. However, two patients with peripheral T cell lymphoma were correctly diagnosed on FDG-PET/CT in spite of low FDG uptake. One patient had enlarged lymph node, larger than 3 cm, in the left inguinal region. Another patient had multiple lymphadenopathy with a short axis around 10 mm and splenomegaly with craniocaudal length of 14 cm. These CT findings may have contributed to the correct diagnosis.

IL-2 is produced mainly by activated CD4+ T cells and interacts with its specific, cell surface-bound receptor, followed by several pathological phenomena (13). A soluble form of its alpha subunit is directly released from the surface of neoplastic cells, thus, reflecting tumor bulk, turnover, and activity (13). On the other hand, elevated sIL-2R is found in a variety of autoimmune and inflammatory diseases (Figure 1) (13). Considering our results that showed no significant difference in sIL-2R between the three groups, sIL-2R is not useful for differential diagnosis between lymphoma and other diseases.

Although diffusely increased FDG uptake in the spleen can be observed in various pathologies, one of the representative causes is invasion of hematological malignancy including lymphoma (14). In our study, 10 patients (90.9%) with higher splenic FDG uptake than that in the liver had a confirmed diagnosis of lymphoma. Splenic FDG uptake may be an important indicator of lymphoma. Further prospective studies are warranted to confirm the worth of splenic FDG uptake.

Our study had some limitations. First, this was a retrospective study including a small number of cases. Because of retrospective design, different evaluators participated in writing interpretation reports of each FDG-PET/CT study, and it is unclear how far they had been able to obtain patient information during evaluation. Second, we excluded patients whose final diagnosis was unconfirmed by histopathology. There could be some patients who were suspected as having lymphoma using FDG-PET/CT but did not have tissue confirmation as clinically they were not felt to have malignancies. These two points can be a cause of selection bias. Third, although this point may be most important, patients with lymphoma that evaluators could not suspect as having lymphoma using FDG-PET/CT were not included in this study. Our subjects were biased, to some extent, toward diffuse large B cell lymphoma, which generally shows extremely elevated FDG uptake. Therefore, non-FDG-avid lymphoma, such as low-grade follicular lymphoma or mucosa-associated lymphoid tissue lymphoma, may have been overlooked. Accordingly, we will need to accumulate those cases to improve diagnostic capacity of FDG-PET/CT to detect lymphoma.

## Conclusion

When the degree of suspicion by nuclear medicine physicians is low, the possibility of lymphoma is also low. Although SUV_max_ and serum level of sIL-2R were not useful for the differential diagnosis, elevated splenic FDG uptake suggested diagnosis of lymphoma.
